# Integrated Optical Mach-Zehnder Interferometer Based on Organic-Inorganic Hybrids for Photonics-on-a-Chip Biosensing Applications

**DOI:** 10.3390/s18030840

**Published:** 2018-03-12

**Authors:** Ana R. Bastos, Carlos M. S. Vicente, Rui Oliveira-Silva, Nuno J. O. Silva, Marta Tacão, João P. da Costa, Mário Lima, Paulo S. André, Rute A. S. Ferreira

**Affiliations:** 1 Department of Physics and CICECO—Aveiro Institute of Materials, University of Aveiro, 3810–193 Aveiro, Portugal; rita.bastos@ua.pt (A.R.B.); cvicente@av.it.pt (C.M.S.V.); ruipedro.silva@ua.pt (R.O.-S.); nunojoao@ua.pt (N.J.O.S.); 2 Instituto de Telecomunicações, University of Aveiro, 3810–193 Aveiro, Portugal; mlima@ua.pt; 3 Department of Electronics, Telecommunications and Informatics, University of Aveiro, 3810-193 Aveiro, Portugal; 4 Department of Biology and CESAM, University of Aveiro, 3810–193 Aveiro, Portugal; martat@ua.pt; 5 Department of Chemistry and CESAM, University of Aveiro, 3810–193 Aveiro, Portugal; jpintocosta@ua.pt; 6 Department of Electric and Computer Engineering and Instituto de Telecomunicações, Instituto Superior Técnico, Universidade de Lisboa, 1049-001 Lisbon, Portugal

**Keywords:** photonic, integrated optics, sol-gel, biosensors, *E. coli*, organic-inorganic hybrid

## Abstract

The development of portable low-cost integrated optics-based biosensors for photonics-on-a-chip devices for real-time diagnosis are of great interest, offering significant advantages over current analytical methods. We report the fabrication and characterization of an optical sensor based on a Mach-Zehnder interferometer to monitor the growing concentration of bacteria in a liquid medium. The device pattern was imprinted on transparent self-patternable organic-inorganic di-ureasil hybrid films by direct UV-laser, reducing the complexity and cost production compared with lithographic techniques or three-dimensional (3D) patterning using femtosecond lasers. The sensor performance was evaluated using, as an illustrative example, *E. coli* cell growth in an aqueous medium. The measured sensitivity (2 × 10^−4^ RIU) and limit of detection (LOD = 2 × 10^−4^) are among the best values known for low-refractive index contrast sensors. Furthermore, the di-ureasil hybrid used to produce this biosensor has additional advantages, such as mechanical flexibility, thermal stability, and low insertion losses due to fiber-device refractive index mismatch (~1.49). Therefore, the proposed sensor constitutes a direct, compact, fast, and cost-effective solution for monitoring the concentration of lived-cells.

## 1. Introduction

The development of biosensors is of extreme importance for highly sensitive and fast pathogen detection with a wide range of applications, such as drug discovery, diagnosis, food safety and processing, environmental monitoring, defense, and security, among others [1]. This has led to the production of precise and powerful analytical tools using biological sensing elements, namely biosensors [1]. There are several techniques to detect and quantify biological elements such as electrochemical methods [2], colony biomass measurements [3], direct counts (e.g., using optical microscopy and flow cytometry), and optical measurements (e.g., using optical fibers [4,5] or optical circuits [6]).

Optical biosensors are a powerful alternative to conventional analytical techniques [7], because they allow an easy-to-use, fast, portable, multiplexed, high specificity, high sensitivity, and cost-effective diagnosis [8]. Nevertheless, all laboratory measurements are essentially based on benchtop instruments with a relatively large footprint, which commonly require sample handling (e.g., dilution), that can be particularly cumbersome when pathogenic or anaerobic microorganisms are the targets of the measurement operation [9]. Optical biosensing also have the disadvantage that absorbance values will vary among spectrophotometers, implying that the determination of growth rates and colonies forming units requires calibration and complementary measurements, respectively [10]. These facts pose a challenge to the scientific community, as there is the need to develop compact optical devices easily operated on the bench and in laminar flow chambers or in the field, in order to determine the concentration of biological elements.

In this context, to produce optical biosensors in a cost-effective way, integrated optics technology is an excellent alternative as it allows the incorporation of both passive and active optical components onto the same substrate for the development of minimized compact sensing devices, using the fabrication of multiple sensors on a single chip [1]. Consequently, these devices result in low reagent consumption and short analysis time, opening prospects for point-of-care applications [11]. Integrated optics systems can be tailored for the detection of specific pathogens, such as bacteria or viruses, by label-free techniques [12] or by simply measuring an optical property, such as refractive index or state of polarization. Integrated optics can be implemented using several devices for biosensing, such as interferometers [11], micro-ring resonators [13], waveguide-coupled surface plasmon resonance sensors [14], and planar directional couplers [15], making them ideal for photonics-on-a-chip applications. The detection principle is based on evanescent field detection. In this case, a bio-receptor layer is immobilized onto the surface of a waveguide, and the exposure to the partner analyte produces a biomolecular interaction affecting the guiding properties of the waveguide (refractive index) [16]. The variation of the refractive index can be correlated with the concentration of the analyte as well as with the affinity constant of the interaction, resulting in a quantitative value of the interaction [16].

Bacterial biosensors based on integrated optics have already been demonstrated [17,18,19,20,21,22], and among these, waveguide interferometer biosensors are advantageous combinations of evanescent field and optical phase difference measurement methods [11], which can be produced in several material fabrication platforms.

In this work, we focus on monitoring bacterial specimens’ concentration in a fluid. In the field of pathogen detection, the most reported bacterial detection methods are made for *Legionella*, *Listeria*, *Salmonella*, *Escherichia coli* (*E*. *coli*), and *Campylobacter* [23]. *E. coli* naturally occurs in the intestinal tract of humans and warm-blooded animals and consists of a diverse group of bacteria. Most *E. coli* strains are innocuous and are an actual integral and important part of a healthy intestinal tract. Nonetheless, some are pathogenic, and consequently may cause disease, including severe diarrhea [24].

Featuring the development of low-cost and miniaturized biosensors with improved sensitivity and stability, a window of opportunity opens to develop biosensors based on integrated optics. Therefore, we propose a cost-effective and real-time biosensing technology based on a Mach-Zehnder interferometer (MZI). The device structure was implemented by direct laser writing on the surface of self-patternable di-ureasil films (refractive index sensor), reducing the complexity and cost production as compared with lithographic techniques. This material was also used because it is synthetized at room temperature from high-purity available precursors, decreasing the production cost and expanding the scope of fabrication availability. In what concerns the optical analysis, we studied the variation of the output MZI intensity—a simpler methodology when compared with spectral analysis, which requires more expensive equipment. The proposed sensor has a low waveguide refractive index contrast with typical values of W_∆N_~10^−4^ [25], avoiding the drawback of ambiguity on a broad measurement range associated with higher refractive index contrast (W_∆N_ = 0.5 [11]) MZI-based sensors [26]. The device was designed to measure in real time the fluid refractive index in the range of 1.320 to 1.380, which is characteristic for some biological fluids, namely *E. coli* cells [27], with a sensibility of 2 × 10^−4^ RIU.

## 2. Experimental Details

### 2.1. Materials Synthesis and Thin Film Deposition

The organic-inorganic hybrids (termed di-ureasils) modified by zirconia-based clusters and methacrylic acid were prepared as described elsewhere [28]. The di-ureasil hybrids were obtained by the hydrolysis and polycondensation of an organic-inorganic triethoxysilane precursor in the presence of zirconium propoxide (Zr(OPr^n^)_4_, Aldrich, 70 wt.% in 1-propanol) and methacrylic acid (McOH, Aldrich 99%), and dispersed in butanol. The amount of Zr(OPr^n^)_4_ was 40% mol with a Zr(OPr^n^)_4_:McOH molar ratio of 1:2 [28]. The sols were processed as films in oxidized silicon substrates (Silicon Quest International; SiO_2_ thickness of 5.00 ± 0.01 µm) during the early stage of gelation via spin-coating (SPIN150-NPPAPT) at 1000 rpm.s^−1^ for 60 s. The films were dried over 24 h at 80 °C for complete solvent removal and densification, leading to a pinhole- and crack-free layer.

### 2.2. Fabrication of the MZI Optical Sensor

**Figure 1:**
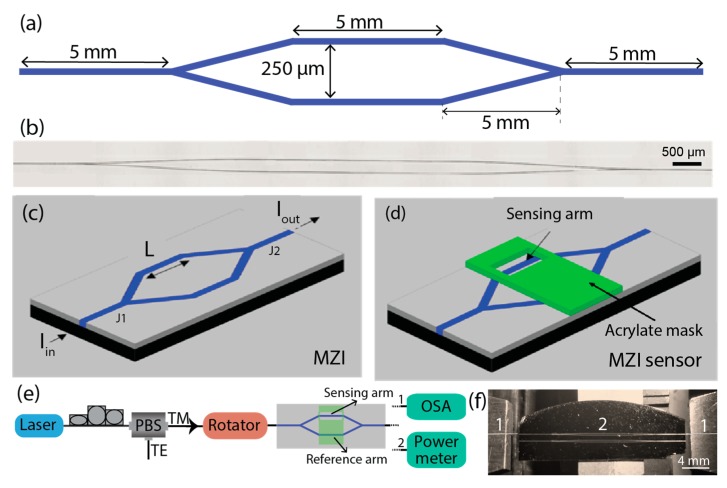
(**a**) General view and detailed dimensions of the Mach-Zehnder interferometer (MZI); (**b**) optical image showing the MZI structure patterned on the surface of a di-ureasil film; (**c**,**d**) scheme of the bare biosensor before and after the liquid container, respectively; (**e**) experimental setup and (**f**) photography of the alignment system used for the optical measurements of the MZI: (1) the optical fibers; (2) MZI patterned region.

The MZI structure is formed by one input/output waveguide with a length of 5 mm and two symmetric Y-junctions with a branch separation of 250 µm and a length of 5 mm. The two Y-junctions are connected by linear waveguides with a length of 5 mm (Figure 1a). The MZI was patterned on the surface of a di-ureasil film (Figure 1b) using a pulsed laser (Coherent Bragg-Star Industrial V2.0), operating at 248 nm with a frequency of 900 Hz and energy of (3.2 − 4.5) × 10^−6^ J per pulse, focused through an objective lens (Thorlabs, LMU-15X-248). The film was moved by a digitally controlled double axis translation system (Newport, XPS and MFA-CC) with a translation velocity of 0.1 × 10^−3^ m·s^−1^. After the patterning process, the silicon wafer substrate was cleaved to allow fiber butt coupling to the device, Figure 1c.

In order to establish a liquid container in one arm of the MZI, a mask of acrylate was used to establish a liquid container in one arm of the MZI. The container aperture has a length (L) of 5 mm, a thickness of ~62.5 µm, and width of 125 µm, and was produced by laser ablation of the acrylate mask in a glass substrate using an UV laser, operating at 300 Hz, with a pulse energy of 1.6 × 10^−3^ J per pulse and translation velocity of 0.1 × 10^−3^ m·s^−1^. After laser ablation, the container was removed from the glass substrate, aligned, and fixed into the optical chip, Figure 1d.

### 2.3. Optical Mode Field Simulation

**Figure 2:**
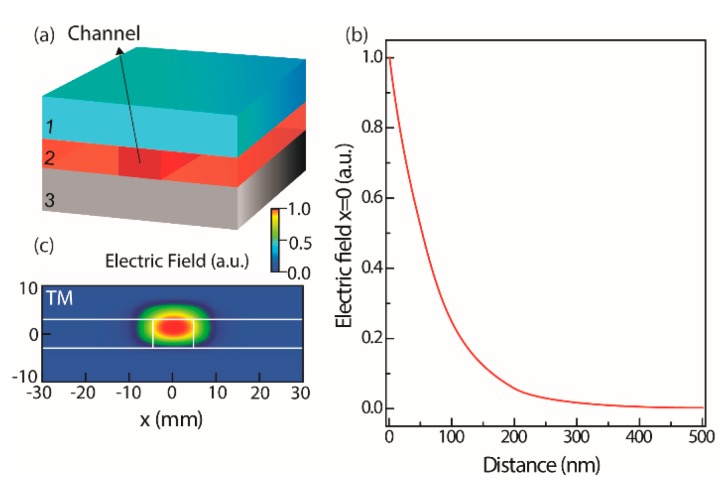
(**a**) Structure model used in the optical mode-simulation, where the layer 1 is the superstrate, 2 is the modified di-ureasil, and 3 is the silica over silicon substrate; (**b**) optical mode field and (**c**) electric field amplitude profile simulation for transverse magnetic (TM) polarization mode.

To estimate the penetration depth of the sensing field, the optical modal analysis at 637 nm in the MZI was performed with the beam propagation method (OptiBPM Designer 9.0, Optiwave^®^). The structure model consists of a stack of three layers, Figure 2a. The hybrid layer has a thickness of 8.0 μm and refractive index values of 1.5040 and 1.5085 outside the channel and in the waveguide region, respectively [25,29]. The silica layer is described by thickness and refractive index values of 5.0 μm and 1.452, respectively, in accordance with the manufacturer. The silicon layer below the silica was not take in consideration, since the propagated optical field has a greatly reduced penetration depth in the silica region, Figure 2c. In the case of the *E. coli* solution, the refractive index value of 1.331 was considered [27,28]. The penetration depth of the sensing field is defined by the exponential decay of the evanescent field while it penetrates into the superstrate medium. Considering the values of the superstrate refractive index for *lysogeny* broth (LB) and *E. coli*, the penetration depth of the sensing field is ~76 nm, Figure 2b. Due to the small penetration of the evanescent field, the sensor detects changes only on the surface of the waveguide, and for that reason any change in the bulk solution will hardly affect the sensor response [16].

### 2.4. E. coli Cell Culture and Refractive Index Measurements

*Escherichia coli* (ATCC 25922) was cultured aerobically in LB at 37 °C. After preparation, the growth medium was autoclaved at 121 °C for 20 min for sterilization. Subsequently, stationary suspensions of *E. coli* were prepared in a laminar air flow chamber. Proliferation was assessed spectrophotometrically by reading the media’s optical density at 550 nm [30]. The refractive index measurements for all liquid media were carried out at 25 °C on an Abbe refractometer (Anton Paar, Abbemat 200) at a wavelength of 589 nm. A drop of *E. coli* suspension (0.2 mL) was placed on the refractometer and the measurements took place until complete evaporation was achieved. The Abbe refractometer was also used to characterize the acrylate-based sample container.

### 2.5. Optical Sensor Measurements

The scheme of the experimental setup used to optically characterize the MZI optical sensor is displayed in Figure 1e. The optical characterization intends to obtain the MZI sensor features and relates them to the refractive index of the *E. coli* solution. The optical signal source was a laser (Agere Systems, SL980S33C) peaking at 980 nm. The polarization selection was achieved through a polarization controller (PC) and a polarization beam splitter (PBS) connected to the MZI sensor with a pigtailed single-mode polarization maintaining (PM) optical fiber (Thorlabs, P1-630PM-FC-1). The optical signal was injected by aligning the PM fiber on a fiber rotator (Thorlabs, HFR007) with a three-dimensional (3D) positioning system (Thorlabs, Nanomax-TS), Figure 1f. The optical signal at the MZI sensor output was recovered with an identical process and the output optical signal was measured with an optical power meter (Noyes, OFM OPM4) with a resolution of 0.05 dB. Data acquisition was done with a sampling time of 0.5 s, beginning with the drop-dispensing process of the fluid containing LB medium and *E. coli* cells on the sensing arm. The transmission spectra of the MZI was also acquired with an optical spectrum analyzer (OSA-EXFO, FTB-500), using an incident signal from an ASE source (Fitel). All optical measurements were independently performed 10 times to monitor the growth process for *E. coli* bacteria and guarantee the experimental repeatability.

## 3. Background and Fundaments

The optical losses (α), defined as the intensity ratio between the output (*I_out_*) and the input (*I_in_*) optical signals (Figure 1c) of an MZI, are given by Equation (1):

(1) $$\alpha (dB)=10\times \log_{10}\left(\frac{I_{out}}{I_{in}}\right)=10\times \log_{10}\left[\frac{1}{2}\left(1+\cos\left(\frac{2\pi \cdot \Delta n_{RS}\cdot L}{\lambda }\right)\right)\right]$$

where λ is the wavelength, *L* is the propagation length, and $∆n_{RS}=n_{R}-n_{S}$ is the difference between the effective refractive index of the reference arm (*n_R_*) and that of the sensing arm (*n_S_*) [16], Figure 1d. The *n_R_* value is constant since the waveguide medium will not change, including the superstrate, an acrylate mask with a refractive index of 1.4740 ± (5 × 10^−4^). The *n_S_* values will show a temporal dependence because the superstrate of the sensor arm will be the fluid formed by the LB medium and *E. coli* cells, whose concentration will increase over time. Therefore, *n_S_* will depend on the refractive index of the fluid (*n_F_*).

**Figure 3:**
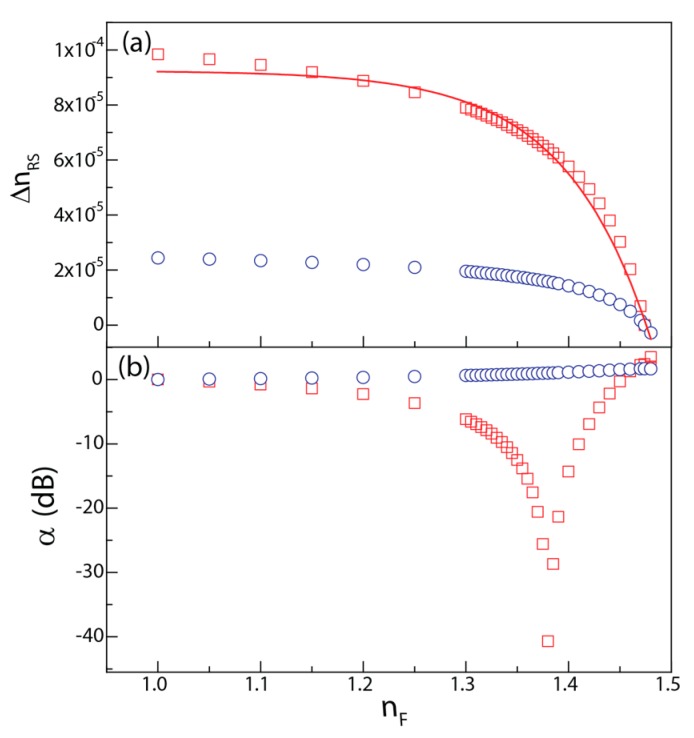
(**a**) ∆*n_RS_* and (**b**) α as a function of *n_F_* for TE (○) and TM polarizations (□). The red line represents the data-best fit (r^2^ > 0.99) for 1.000 ≤ *n_F_* ≤ 1.500 (characteristic for air and *E. coli* cells [32]), using a single exponential decay ($∆n_{RS}=y_{0}+A\times e^{\left(-n_{F}/t\right)}$ , with *y_0_* = (9.3 ± 0.1) × 10^−6^, *A* = −1.9 × 10^−12^ and *t* = −8.40 ± 0.04 × 10^−2^).

To estimate ∆*n_RS_*, for transverse electric (TE) and transverse magnetic (TM) polarizations, the embedded waveguide (3D) was modeled as an asymmetric slab waveguide (two-dimensional (2D)). This approximation is valid since the channel was patterned by UV-direct laser writing, which induces a low refractive index increase (~10^−4^) [25] in the UV-exposed region (channel region) compared with the non-exposed one. The waveguide propagation equation [31] was solved with an analytic method [32] for the interval 1.000 ≤ *n_F_* ≤ 1.500, Figure 3a. Based on these results, α was calculated as a function of *n_F_* using Equation (1), for TE and TM polarizations, Figure 3b. Whereas the α values are almost constant for TE polarization, for TM polarization α reveals an asymptotic decay (~40 dB, when *n_F_* ≈ 1.380), ascribed to the increase of the evanescent field on the interface film/superstrate [33]. The MZI output is governed by a sinusoidal function (Equation (1)), revealing an asymptotic response when the cosine argument tends to π + 2kπ (with *k* = 0, 1, 2, …). Applying Equation (2), *n_F_* was estimated for α values in TM polarization through:

(2) $$n_{F}=-t\times ln\left(\frac{\frac{\lambda }{2\pi L}acos\left(2\times 10^{\propto /10}-1\right)-y_{0}}{A}\right)$$

where *t*, *A*, and *y_0_* are fit parameters of the exponential decay $∆n_{RS}=y_{0}+A\times e^{\left(-n_{F}/t\right)}$.

The fluid was described by a mixture of two components (designated as A and B). During the measurement interval (~800 s), the number of *E. coli* living cells is constant, as the *E. coli* cell-cycle duplication period is ~1 h [34,35]. Thus, *n_F_* can be described by the Lorentz-Lorenz relation [36]:

(3) $$\frac{n_{F}^{2}-1}{n_{F}^{2}+2}=\phi_{A}\frac{n_{A}^{2}-1}{n_{A}^{2}+2}+\phi_{B}\frac{n_{B}^{2}-1}{n_{B}^{2}+2}$$

where *φ_A_* and *φ_B_* are the volume fractions of the two components, and *n_A_*, *n_B_* are the refractive indexes. Considering that component A is the LB medium and component B is the *E. coli* cells, the volume of *E. coli* cells in the fluid is defined by *V_EC_* = *φ_B_ × V_total_*. Therefore, the number of *E. coli* cells is *N* = *V_EC_*/*V_cell_*, where *V_total_* and *V_cell_* are the volumes of the fluid and the individual cells, respectively. The *V_cell_* can be approximately defined as a cylinder with a length of 2 µm and a width of 1 µm, and the mass of each cell is 1 pg [37]. The *E. coli* cells concentration (*C*) can then be estimated by Equation (4):

(4) $$C=\frac{\phi_{B}}{V_{cell}}$$

In order to evaluate the sensor performance, the error associated with the calculation of *n_F_* was calculated through:

(5) $$∆n_{F}=\left|\frac{\partial n_{F}}{\partial \alpha }\right|∆\alpha =\frac{∆\alpha \times ln\left(10\right)\times t}{10\times acos\left(2\times 10^{\alpha /10}-1\right)}\sqrt{\frac{1}{10^{-\alpha /10}-1}}$$

where ∆α = 0.05 dB is the optical power meter experimental resolution.

Combing Equations (2), (3), and (5), the error in the concentration value (limit of detection, LOD) can be calculated through:

(6) $$LOD=\frac{∆n_{F}}{V_{cell}}\frac{n_{B}^{2}+2}{n_{B}^{2}-1}\left[\frac{6n_{F}}{{\left(n_{F}^{2}+2\right)}^{2}}-\phi_{A}\frac{n_{A}^{2}-1}{n_{A}^{2}+2}\right]$$

## 4. Results and Discussion

**Figure 4:**
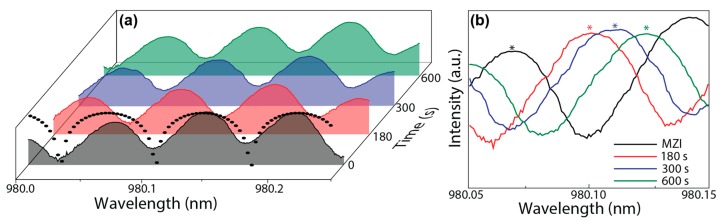
(**a**) Simulated (solid circles) and experimental transmission spectra of the MZI and the respective temporal dependence after the fluid spread in the sensing region for selected time intervals; (**b**) magnification of the spectral region used to estimate *n_F_*.

Figure 4 shows the transmission spectrum of the MZI output, revealing a typical sinusoidal interference pattern, as described by Equation (1). After the *E-coli*-based fluid spread in the sensing region, the transmission spectra revealed a red-shift that increased with time, Figure 4b, which is related to the increase of *n_F_*, as detailed below. Further evidences of the *n_F_* temporal dependence can also be found through the study of the variation of the intensity ratio between *I_out_* and *I_in_*, which is a simpler methodology when compared with spectral analysis.

**Figure 5:**
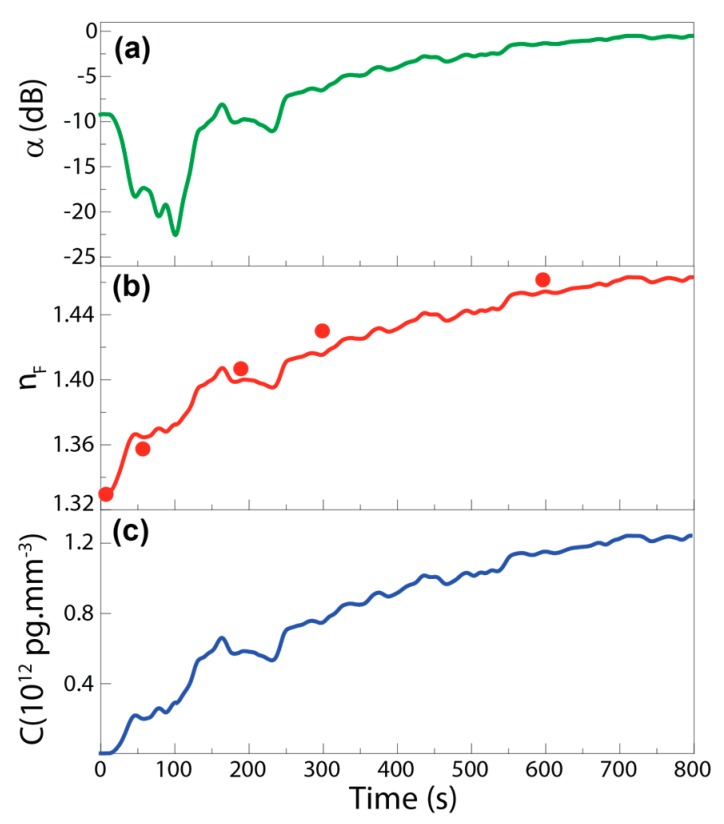
Temporal evolution of the (**a**) MZI sensor optical power loss, (**b**) *n_F_*, and (**c**) *E. coli* concentration. The solid circles in (**b**) correspond to the values estimated by the spectral shift analyses.

The optical loss α was measured for TM polarization, since α revealed an asymptotic decay with *n_F_* for this polarization. Figure 5a shows the temporal evolution of the optical signal loss, after the spread of the fluid in the sensing region and signal stabilization (α ≈ −10 dB). During the measurements, the water from the LB medium evaporated, leading to an increase of *E. coli* concentration until the complete evaporation of water in the LB medium was achieved. During this process the fluid refractive index increased towards a final value that is closer to the *E. coli* refractive index. Evaluating α temporal dependence, a minimum value was observed at ~−23 dB, corresponding to the MZI asymptotic response that occurred for *n_F_* = 1.38 (represented in Figure 3b). The subsequent increase to ~−0.5 dB occurred for *n_F_* > 1.38. To guarantee a univocal sensor response, the asymptotic region must be towards higher *n_F_* values; for instance, by tailoring the length of the sensing arm, if *L* = 7 µm, the measurement range can be extended for 1.00 < *n_F_* < 1.43 [16].

By analyzing α and applying Equation (1), *n_F_* was determined, Figure 5b. The initial value of the fluid refractive index was 1.3310 ± (2 × 10^−4^), and during the water evaporation, *n_F_* changed until a maximum value of 1.4632 ± (2 × 10^−4^) that was recorded at ~800 s. This *n_F_* variation can be rationalized through the calculation of weight average value between *n_EC_* (~1.388) and *n_LB_* (~1.330) [27,38]. In the beginning of the experiment, the main contribution for *n_F_* derived from the water in the LB growth medium, and after the evaporation, the main contribution derived from the *E. coli* cells as well as fats and proteins left from the LB (*n_fats_* = 1.40–1.50, [39]), which led to a *n_F_* value close to those of *n_EC_* and *n_fats_*. At *t* = 100 s a *n_F_* variation of (+4.15 ± 0.02) × 10^−2^ was observed, which closely matched the variation measured by the Abbe refractometer in the same conditions, (+4.20 ± 0.05) × 10^−2^, validating the results obtained by the MZI sensor. The *n_F_* variation was also estimated through the transmission spectra in Figure 4. In particular, the temporal dependence of the peak position around 980 nm was used to obtain *n_F_* through Equation (1). As illustrated in Figure 5b (solid circles) the estimated values are also in good agreement with those predicted by the intensity analysis. The error associated with these calculations is lower than 10^−6^ RIU, considering that the main error is the experimental resolution of the optical spectrum analyser (0.8 pm).

For biosensing, in particular to monitor the concentration growing of bacteria in a medium and estimate the sensor figures, the α temporal variation was used to calculate *C* through Equation (3), Figure 5c. The initial concentration (*C_0_*) was estimated as ~2.93 × 10^9^ cells·mL^−1^, which is a typically reported value for *E. coli* after overnight growth in a rich medium, such as LB [27]. When the water evaporation occurred, *C* reached a stable value of ~1.23 × 10^12^ cells·mL^−1^.

In order to evaluate the sensor performance, the refractive index sensitivity (∆*n_F_*) was calculated, considering that the main error is associated with the estimation of α, which was assumed as the error of the optical power meter (0.05 dB), yielding to ∆*n_F_* ≤ 2 × 10^−4^ RIU, which is the best-value reported for low-refractive index contrast biosensors. To the best of our knowledge, larger or analogous values were only achieved for silicon-based devices, which use more complex and expensive photolithographic techniques compared with direct-lased writing, Table 1. We note that the RIU value is also comparable with the figure of merit known for a silicon-on-insulator MZI for sensing using the spectral shift methodology (e.g., RIU~10^−4^ estimated for a typical experimental spectral resolution of 10^−11^ m) [40].

Therefore, we compare our results with the figures of merit known for polymer-based sensors, whose ∆*n_F_* is larger (3 × 10^−3^ RIU) for a similar refractive index contrast (W_∆N_ < 10^−3^) [40]. Based on the estimated RIU values, a maximum LOD for the proposed sensor was calculated using Equation (6), yielding 2.0 pg·mm^−3^ (2.0 × 10^3^ cells/mL), which is among the best values reported in the literature (Table 1).

With a balance between the optimized measure window targeted to quantify the refractive index of biological fluids, sensitivity, and LOD, the proposed sensor is predicted to be able to measure and detect biological events such as bacterial growth [2,27,38] and hemoglobin concentrations [46], as well as monitor high cell density fermentation processes [47].

## 5. Conclusions

This manuscript describes the design and experimental demonstration of a cost-effective optical biosensor for the determination of *E. coli* concentration, based on an MZI structure fabricated by laser direct writing on a self-patternable di-ureasil organic-inorganic film. The sensor was designed using analytic models in order to establish the optical behavior of the device, envisioning the desired application. The *E. coli* concentration was measured during an induced evaporation process of the medium from 2.93 × 10^9^ to ~1.23 × 10^12^ cells·mL^−1^ within a few minutes. The sensibility and LOD of the sensor were estimated to be 2 × 10^−4^ RIU and 2.0 pg·mm^−3^, respectively, which are record values known for integrated optics-based solutions with low refractive index contrast. Featuring enhanced values, the sensor sensitivity and LOD can be easily improved (∆*n_F_* = 2 × 10^−5^ RIU and LOD = 0.1 pg·mm^−3^) by using a higher resolution power meter (e.g., 0.001 dB) or through spectral analysis (≤10^−6^ RIU), despite the higher cost. The use of such a simple design and the exploitation of a low contrast fabrication platform allowed the development of an optical biosensor with a relatively simple fabrication procedure and interrogation scheme, and without the drawback of the ambiguity on a broad measurement range (1.00 ≤ *n_F_* ≤ 1.38) associated with high-resolution MZI-based sensors. The interrogation scheme reported here can also be simplified by introduction on the optical chip of thermo-optic polarization-controlling elements, already demonstrated with this photo-patternable material, which can ultimately lead to a more compact and portable device.

## Figures and Tables

**Table 1:** Refractive index contrast, sensitivity (RIU), and limit of detection (LOD, pg·mm^−3^) of selected MZI optical biosensors using the intensity methodology. The method used to fabricate the sensor is also indicated.

Material	Method	W_∆N_	RIU	LOD	Reference
di-ureasil	Direct writing	10^−4^	2 × 10^−4^	2.0	Present work
SiO_2_		5 × 10^−3^	1.5 × 10^−4^	-	[41]
ORMOCER^®^	Photolithography	-	10^−5^	2.4	[42]
Si-based		-	2 × 10^−4^	-	[43]
SiN		-	3 × 10^−3^	-	[44]
PDMS		-	-	2	[45]

ORMOCER^®^ = OrganicModified Ceramic − Fraunhofer ISC; PDMS = Polydimethylsiloxane.
